# Single-cell RNA-seq analysis reveals the Wnt/Ca^2+^ signaling pathway with inflammation, apoptosis in nucleus pulposus degeneration

**DOI:** 10.1186/s12891-024-07368-3

**Published:** 2024-04-23

**Authors:** Peigeng Wang, Zhencong Li, Dongping Ye

**Affiliations:** 1 Guangzhou Red Cross Hospital, Guangzhou Red Cross Hospital of Jinan University, Guangzhou, Guangdong Province 510220 China; 2 Department of Spinal Degeneration and Deformity Surgery, Affiliated Hospital of Guangdong Medical University, Zhanjiang, Guangdong Province 524001 China; 3 Guizhou Medical University, Guizhou Medical University, Guiyang, Guizhou Province, 550025 China

**Keywords:** Single cell sequencing, Wnt/Ca^2+^ signaling pathway, Disc degeneration, Nucleus pulposus, Inflammation, Apoptosis

## Abstract

**Background:**

Increasing studies have shown degeneration of nucleus pulposus cells (NPCs) as an critical part of the progression of intervertebral disc degeneration (IVDD). However, there are relatively few studies on single-cell transcriptome contrasts in human degenerated NPCs. Moreover, differences in Wnt/Ca^2+^ signaling in human degenerated nucleus pulposus cells have not been elucidated. The aim of this study is to investigate the differential expression of Wnt/Ca^2+^ signaling pathway between normal and degenerated nucleus pulposus cells in humans and try to investigate its mechanism.

**Methods:**

We performed bioinformatics analysis using our previously published findings to construct single cell expression profiles of normal and degenerated nucleus pulposus. Then, in-depth differential analysis was used to characterize the expression of Wnt/Ca^2+^ signaling pathway between normal and degenerated nucleus pulposus cells in humans.

**Results:**

The obtained cell data were clustered into five different chondrocytes clusters, which chondrocyte 4 and chondrocyte 5 mainly accounted for a high proportion in degenerated nucleus pulposus tissues, but rarely in normal nucleus pulposus tissues. Genes associated within the Wnt/Ca^2+^ signaling pathway, such as Wnt5B, FZD1, PLC (PLCB1), CaN (PPP3CA) and NAFATC1 are mainly present in chondrocyte 3, chondrocyte 4 and chondrocyte 5 from degenerated nucleus pulposus tissues. In addition, as a receptor that activates Wnt signaling pathway, LRP5 is mainly highly expressed in chondrocyte 5 of degenerated nucleus pulposus cells. Six genes, ANGPTL4, PTGES, IGFBP3, GDF15, TRIB3 and TNFRSF10B, which are associated with apoptosis and inflammatory responses, and are widespread in chondrocyte 4 and chondrocyte 5, may be closely related to degenerative of nucleus pulposus cells.

**Conclusions:**

Single-cell RNA sequencing revealed differential expression of Wnt/Ca^2+^ signaling in human normal and degenerated nucleus pulposus cells, and this differential expression may be closely related to the abundance of chondrocyte 4 and chondrocyte 5 in degenerated nucleus pulposus cells. In degenerated nucleus pulposus cells, LRP5 activate Wnt5B, which promotes nucleus pulposus cell apoptosis and inflammatory response by regulating the Wnt/Ca^2+^ signaling pathway, thereby promoting disc degeneration. ANGPTL4, IGFBP3, PTGES in chondrocyte 4 and TRIB3, GDF15, TNFRSF10B in chondrocyte 5 may play an important role in this process.

## Introduction

Intervertebral disc degeneration (IVDD) is a degenerative disease that often occurs in the middle-aged and elderly population and has been widely considered as one of the important triggers of low back pain. IVDD can not only lead to severe nerve damage and disability, but also a major cause of chronic pain and degenerative diseases of the spine, bringing a huge financial burden to individuals, families and society [1, 2]. The etiology of IVDD is complex and its pathogenesis remains incompletely understood. Aging, genetic factors, and inflammation are all associated with the development and progression of IVDD [3]. Clinically, histopathological and radiographic data suggest that most low back pain begins with degenerative changes in the nucleus pulposus of the intervertebral disc [4]. Anatomically, the intervertebral disc (IVD) is a soft connective tissue connecting the adjacent vertebral bodies of the spine and an avascular tissue that consists mainly of the central glial nucleus pulposus (NP), peripheral annulus fibrosus (AF), and cartilage endplate (CEP). The IVD has multiaxial flexibility to transfer and buffer spinal pressures caused by body weight and muscle contraction [5]. Nucleus pulposus (NP) consists of type II collagen, proteoglycan, and nucleus pulposus cells. NP cells play a key role in maintaining disc integrity as it produces aggregate proteoglycans and extracellular matrix (ECM) components such as type II and X collagen [6]. Increasing evidence suggests that abnormal function of nucleus pulposus cells (NPC), including changes in cell proliferation, apoptosis, ECM production/degradation, and cytokine secretion at the mRNA level, is critical for the pathogenesis of IVDD [7]. Thus, degeneration of nucleus pulposus cells (NPCs) is an indispensable part of IVDD progression [8].

The Wnt/Ca^2+^ signaling pathway is an important branch of the Wnt signaling pathway, which signal transduction pathway is closely related to cellular responses caused by developmental, cancer, and inflammatory stimuli [9]. Studies have shown that the synthesis of various growth factor proteins such as Rac1 [10], Periostin [11], TGF-β, and insulin growth factors [12]in the nucleus pulposus can be induced by regulating the Wnt/β-catenin signaling pathway, thereby accelerating its degeneration process. The relationship between Wnt/β-catenin signaling and apoptosis and inflammatory response has been shown to be closely related [13]. However, up to now, The key genes in Wnt signaling that trigger degenerative NPCs degeneration-related signaling pathways, apoptosis and inflammation have not been fully elucidated.

In the study of pathogenesis, the most commonly performed research objects in the past are the whole pathological changes of these overall tissues, so there are many limitations and inaccuracies in the previous research methods, such as ignoring the role of the heterogeneity of individual cells inside some disease tissues in these diseases. With advances in scRNA-seq technology, heterogeneous tissues can be delineated at the single-cell level [14]. Single-cell RNA sequencing (scRNA-seq) provides an opportunity to explore heterogeneity and cell-cell interactions in complex tissues at high resolution [15]. However, there are relatively few studies on the comparison of single cell transcriptomes in human degenerated nucleus pulposus tissues. Moreover, the Wnt/Ca^2+^ signaling pathway has not been reported in degenerated nucleus pulposus tissues. Therefore, it is urgent to investigate the cellular heterogeneity between different nucleus pulposus tissues by scRNA-seq, perform a more comprehensive single-cell analysis of nucleus pulposus tissues, determine the genetic characteristics associated with nucleus pulposus cell degeneration, find new targets for the treatment of disc degeneration, and provide a theoretical basis for the clinical treatment of disc degeneration. Our analysis suggests that wnt5B, which is activated by LRP5, promotes apoptosis and inflammatory responses in nucleus pulposus cells by regulating the Wnt/Ca^2+^ signaling pathway, thereby promoting disc degeneration. Some genes within the chondrocytes may play an important role in this process.

## Materials and methods

### Patient and sample collection

To define the heterogeneity of NP-normal and NP-degenerative cells at the transcriptional level, we performed bioinformatic analysis using results obtained from our previously published studies [16]. The datasets presented in this study can be found in online repositories. The names of the repository/repositories and accession number(s) can be found below: https://www.ncbi.nlm.nih.gov/geo/query/acc.cgi?acc=GSE205535. In this study, degenerated nucleus pulposus tissue samples came from an 81-year-old patient diagnosed with lumbar disc herniation/lumbar disc degeneration, and normal nucleus pulposus tissue samples came from an 11-year-old patient diagnosed with acute spinal cord injury. Fresh specimens collected at the time of surgical resection were collected in MACS tissue stock (Miltenyi Biotec, Germany) and sent to the laboratory as soon as possible. Informed consent was obtained from all subjects and/or their legal guardian(s). Patient or family has signed an informed consent. The study was approved by the Guangzhou Red Cross Hospital ethics committee.

### Processing of scRNA-sequencing data

RNA-seq data analysis was performed by NovelBio Bio-Pharm Technology Co. and ltd using the NovelBrain Cloud Analysis Platform. We applied fastp [17] to filter adapter sequences to remove low-quality reads to achieve clean data. Single cell transcriptome analysis was performed using the UMI tool [18] to identify a white list of cell barcodes.

### Primary analysis of raw read data, quality control, dimension-reduction and clustering

Using the Seurat package (version: 3.1.4, https://satijalab.org/seurat/), cell normalization and regression were performed according to expression tables to obtain scaled data based on UMI counts and percentage mitochondrial rate for each sample. Because the samples were batch processed and sequenced, we used MNN (mutual nearest neighbor) to eliminate potential batch effects. Subsequently, the first 10 principles were used for UMAP construction. Using a graph-based clustering approach (resolution = 0.8), unsupervised cell clustering results were obtained according to the first 10 principles, and marker genes were calculated using the FindAllMarkers function and Wilcox rank sum test algorithm with the following calculation criteria: (1) lnFC > 0.25; (2) *p*-value < 0.05; (3) min. PCT > 0.1. To identify cell types in detail, clusters of the same cell type were selected for UMAP analysis, graph-based clustering, and labeling analysis. Variant genes were selected for principal component analysis (PCA) using FindVariableFeautres software, and cells were visualized in two-dimensional space using the tSNE algorithm [19].

### Differential gene identification of the Wnt/Ca^2+^ signaling pathway between normal and degenerative nucleus pulposus Cells (RT-qPCR)

Normal and degenerated nucleus pulposus tissue samples were collected and immediately frozen at 80 °C, and the expression levels of the genes expression in the Wnt/Ca^2+^ signaling pathway were detected by RT-qPCR. Differences in expression of the Wnt/Ca^2+^ signaling pathway between normal and degenerated nucleus pulposus cell populations were verified based on changes in expression levels of related genes.

### Statistical analysis

All statistical analyses and data were generated using R software (version 3.6.3). And calculated two independent samples by Wilcox test. *p*-value < 0.05 was considered statistically significant.

## Results

### Cellular distribution in NP-degenerative and NP-normal

**Fig. 1:**
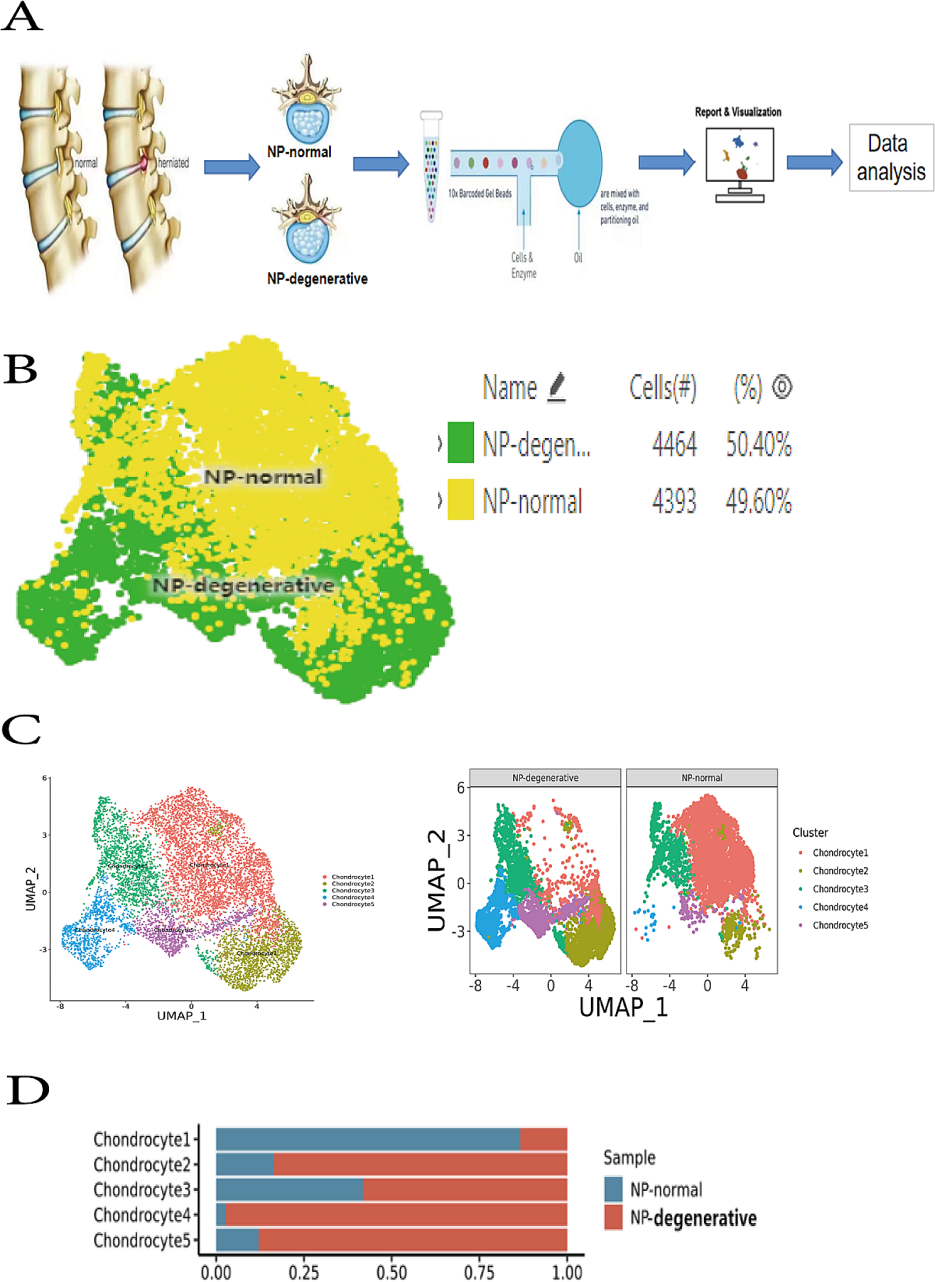
Single cell sequencing demonstrated clusters of chondrocytes from NP-degenerative and NP-normal. (**A**) Schematic of experimental workflow for defining and comparing NP cells between all donor groups. (**B**) UMAP of 8,857 cells profile with each cell color-coded for sample types. (**C**) UMAP of single cell profile with each cell color coded for chondrocyte clusters (left to right): the associated cell type, and merged status. (**D**) The proportion of each cluster

ScRNA-seq analysis of nucleus pulposus tissues from 2 tissuss (Fig. 1A). After filtering, a total of 8,857 chondrocytes were retained for downstream analysis, including 4,464 degenerated nucleus pulposus chondrocytes (NP-degenerative) and 4,393 normal nucleus pulposus chondrocytes (NP-normal) (Fig. 1B). These cells were clustered into five distinct clusters, chondrocyte 1, chondrocyte 2, chondrocyte 3, chondrocyte 4, and chondrocyte 5(Fig. 1C). Among them, chondrocyte 4 and 5 mainly appeared in degenerated nucleus pulposus tissue(Fig. 1D).

### Five Chondrocyte Clusters are Identified by scRNA-Seq in NP-degenerative and NP-normal

The chondrocyte 1 accounted for a higher proportion of normal nucleus pulposus cells compared with degenerated nucleus pulposus cells (Fig. 1D). Overall, the most obvious differences were chondrocyte 4 and chondrocyte 5, specifically, chondrocyte 4 and chondrocyte 5 mainly accounted for a high proportion in NP-degenerative, but a small proportion in NP-normal (Fig. 1C and D).

### scRNA-seq analysis reveals differential expression of the LRP5 in NP-degenerative and NP-normal

**Fig. 2:**
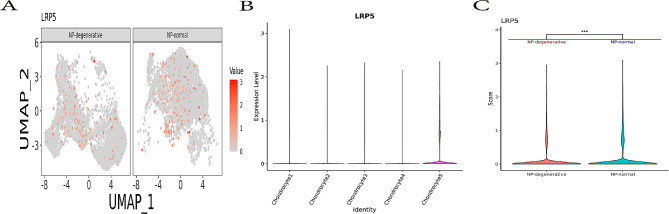
Expression of LRP5 activating Wnt/Ca^2+^ signaling pathway in nucleus pulposus cells. (**A**) UMAP Plot showing the expression of LRP5 in NP-degenerative and NP-normal. (**B**) Violin plot showing the expression of LRP5 in five Chondrocyte clusters. (**C**) Differential expression analysis was performed comparing LRP5 within NP-degenerative and NP-normal. Stars indicate significance level of gene expression difference between two samples by Wilcox test. ***, *p* value < 0.001

Low-density lipoprotein receptor-associated protein 5 (LRP5) is a receptor which be proven activate Wnt signaling. In the results of this study, LRP5 was highly expressed in chondrocyte 5 of NP-degenerative tissues (Fig. 2A and B). Moreover, the expression of LRP5 in NP-degenerative cells and NP-normal cells was significantly different, Stars indicate significance level of gene expression difference between two samples by Wilcox test. *P* value < 0.001 (Fig. 2C).

### scRNA-seq analysis reveals differential expression of the Wnt/Ca^2+^ Signaling Pathway in NP-degenerative and NP-normal

**Fig. 3:**
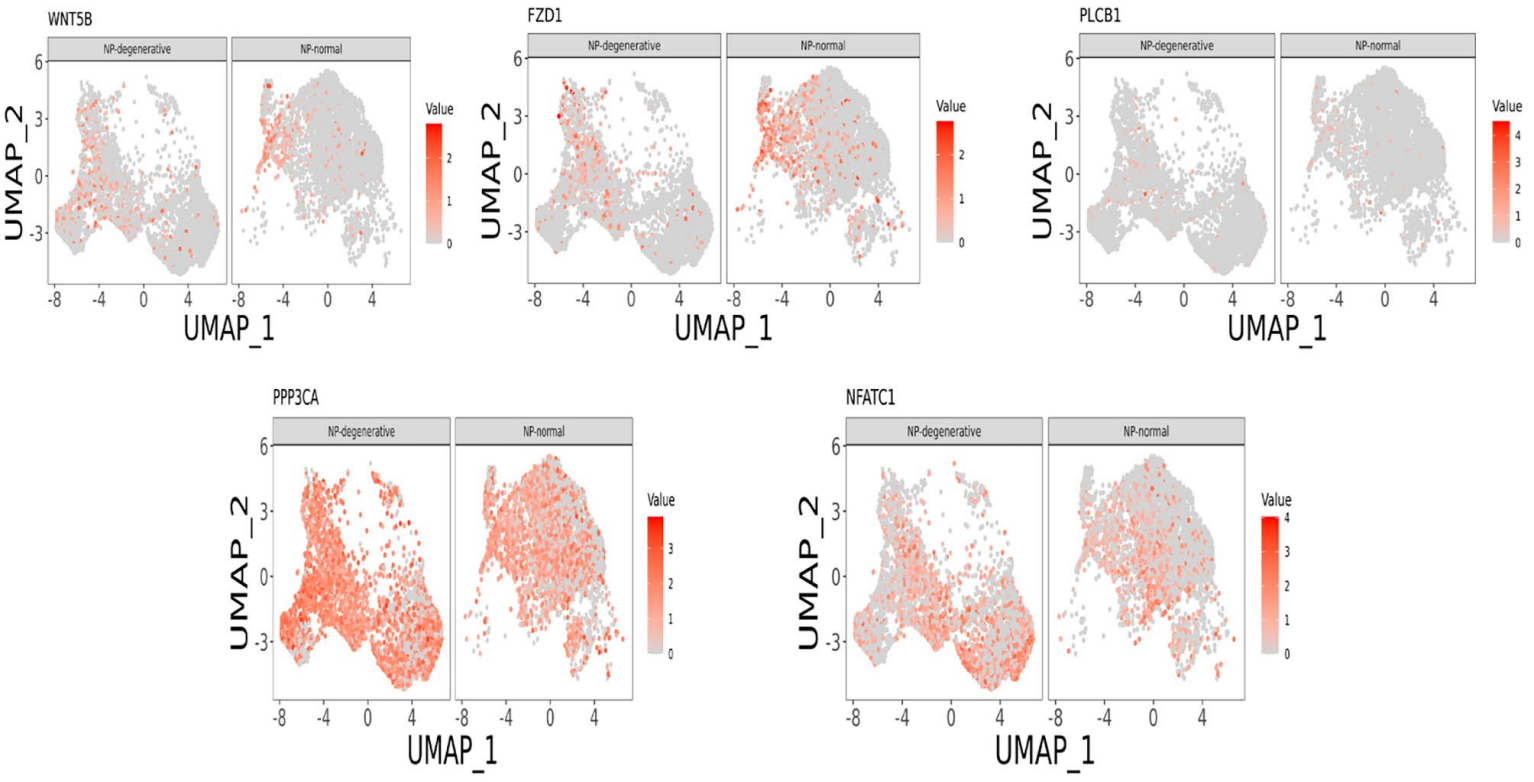
The signature genes within Wnt/Ca^2+^ Signaling Pathway from NP-degenerative and NP-normal, embedded on UMAP dimension reduction map, and colored by gene expression levels

**Fig. 4:**
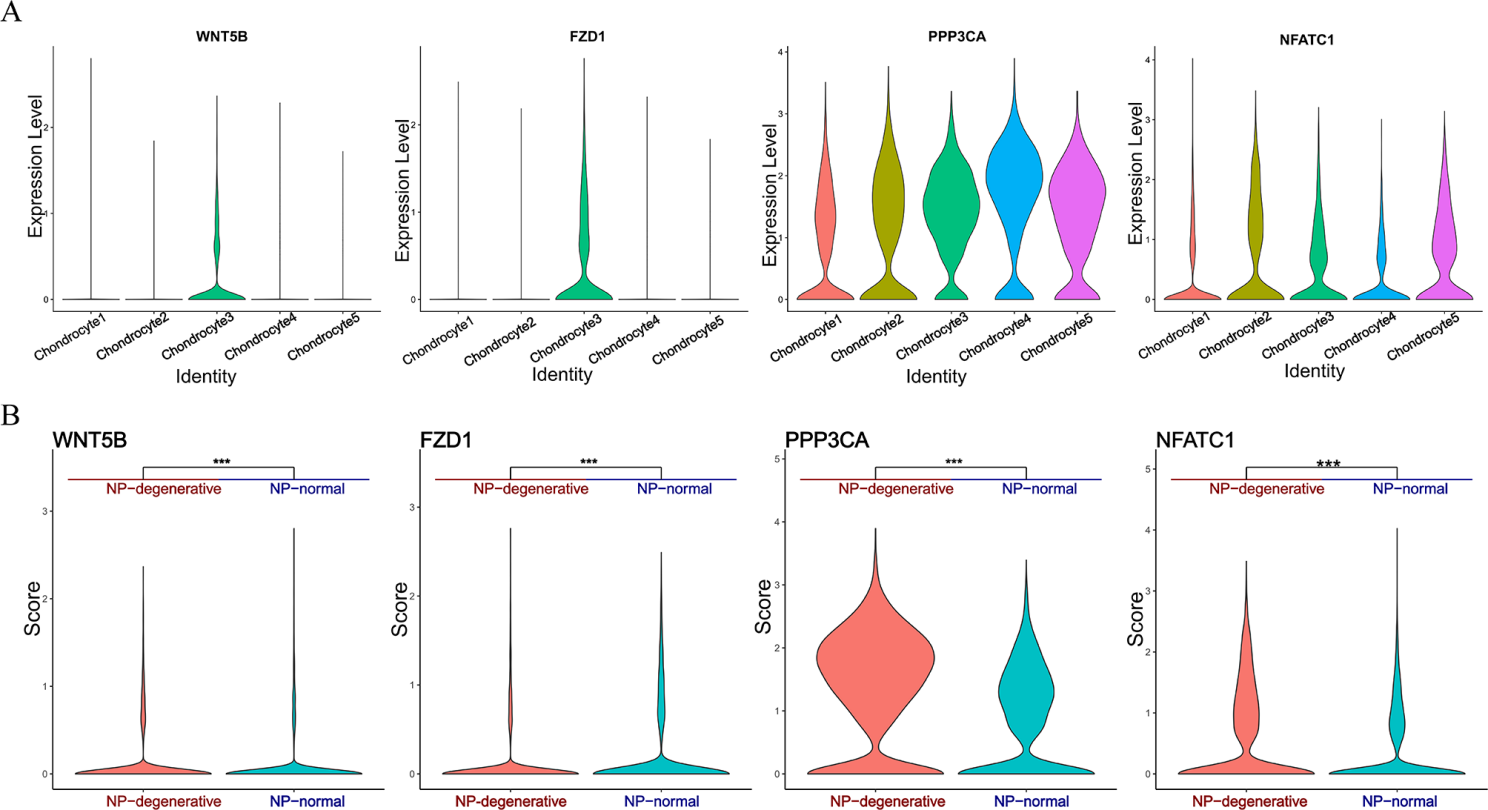
Expression of marker genes within Wnt/Ca^2+^ Signaling Pathway. (**A**) Violin plot showing scaled expression of differentially expressed genes defining the five chondrocyte clusters. (**B**) Differential expression analysis was performed comparing marker genes within Wnt/Ca^2+^ Signaling Pathway from NP-degenerative and NP-normal. Stars indicate significance level of gene expression difference between two samples by Wilcox test. ns, not significant; *, *p* value < 0.05; **, *p* value < 0.01; ***, *p* value < 0.001

Wnt5B, FZD1, PLC (PLCB1), CaN (PPP3CA), and NAFAT (NAFATC1) within the Wnt/Ca^2+^ signaling pathway were found to be significantly different in five different chondrocyte clusters with NP-degenerative and NP-normal (Fig. 3 and Fig. 4). To be precise, although Wnt5B is expressed in both NP-degenerative and NP-normal cells, compared with NP-normal cells, Wnt5B is mainly enriched in chondrocytes 3, chondrocytes 4 and chondrocytes 5 of NP-degenerative cells, and lesser in other clusters. At the same time, the expression of FZD1, PLC (PLCB1), CaN (PPP3CA), and NAFAT (NAFATC1) all have similar differences as Wnt5B (Fig. 3** and** Fig. 4).

**Fig. 5:**
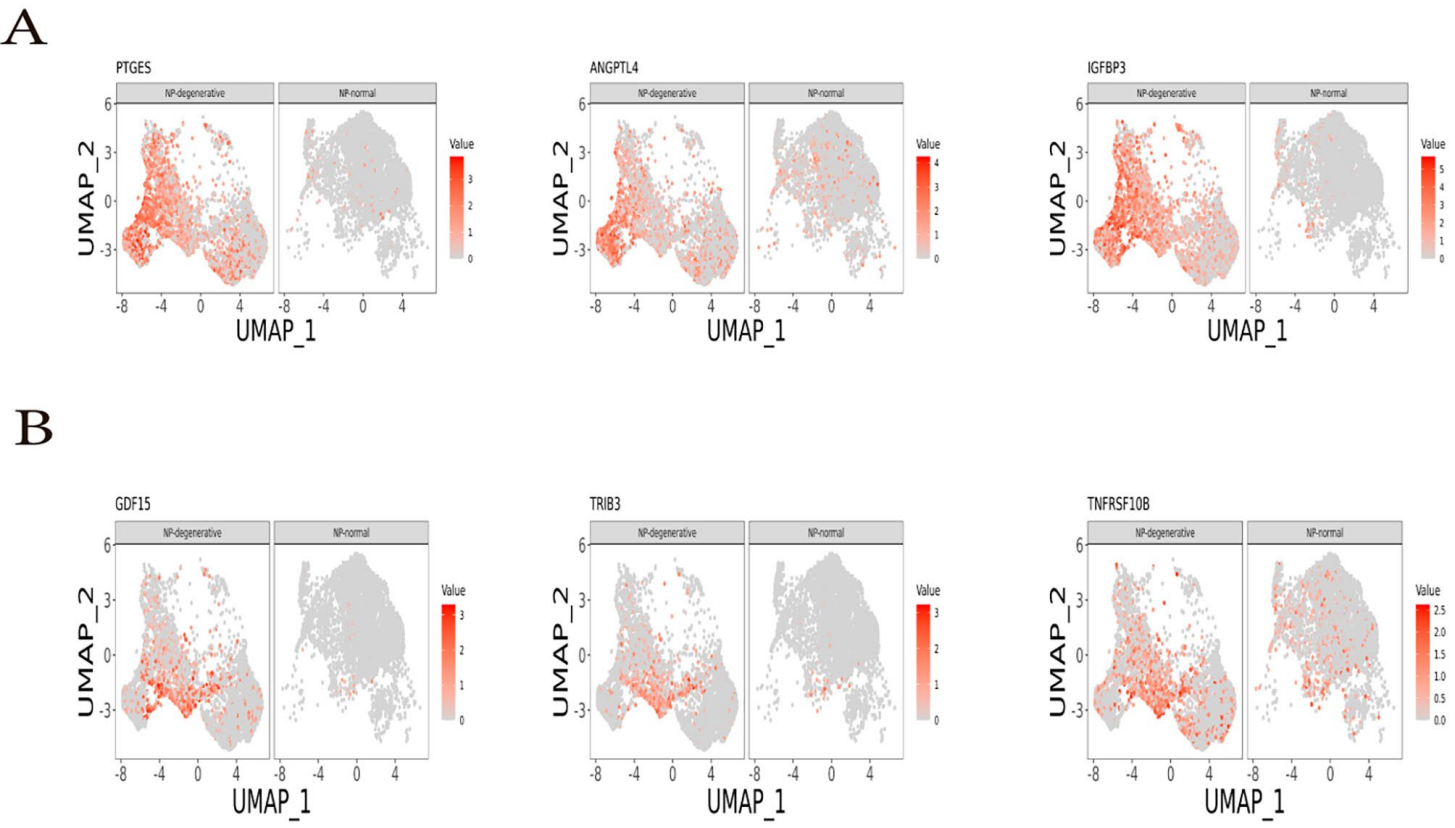
The differentially expressed genes of NP-degenerative and NP-normal, embedded on UAMP dimension reduction map, and colored by gene expression levels. (**A**) UAMP Plot showing PTGES, ANGPTL4 and IGFBP3 are mainly highly enriched in chondrocyte 4 of NP-degenerative. (**B**) UAMP Plot showing GDF15, TRIB3 and TNFRSF10B are mainly highly enriched in chondrocyte 5 of NP-degenerative

**Fig. 6:**
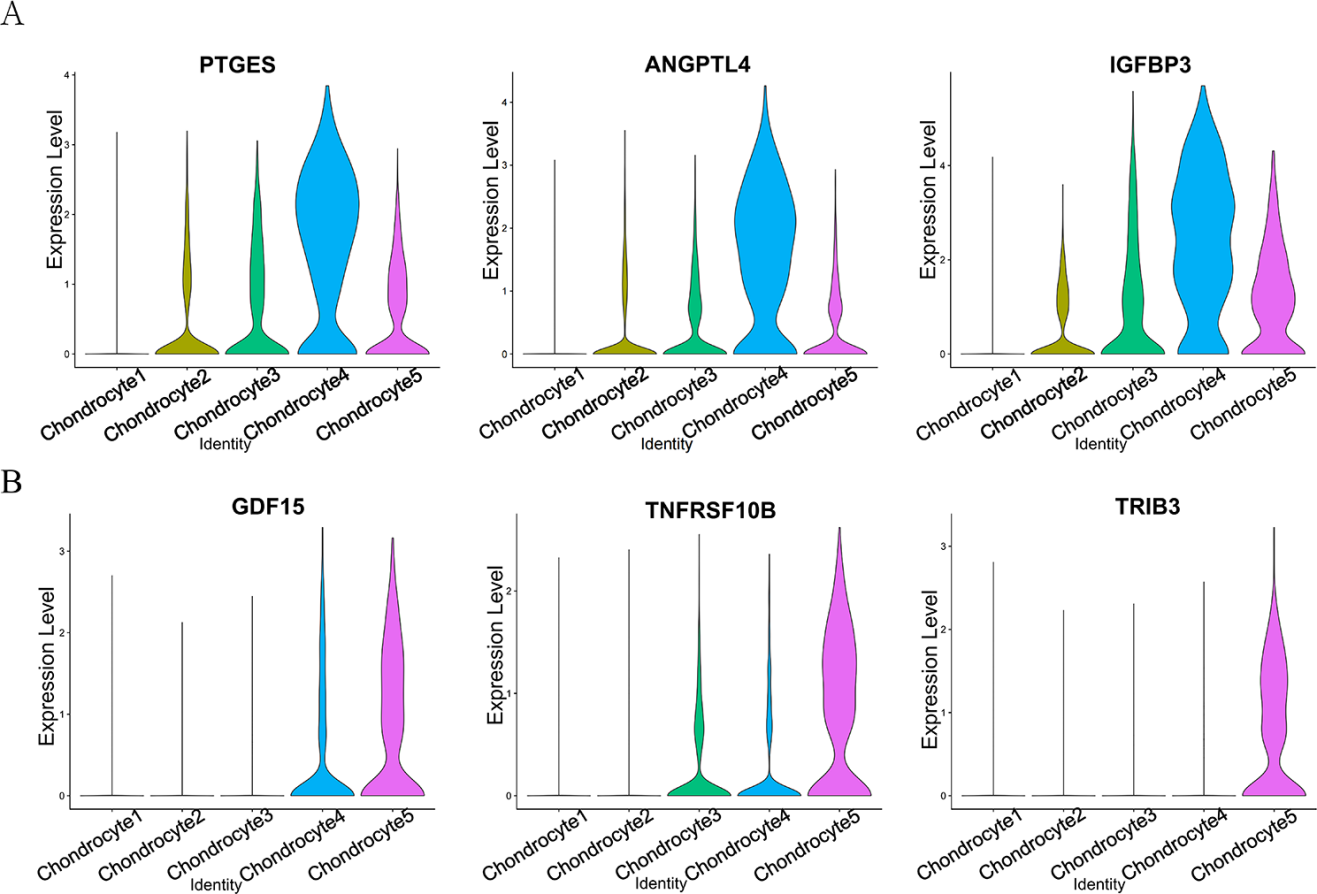
Expression of the marker genes. (**A**) Violin plot showing PTGES, ANGPTL4 and IGFBP3 are mainly highly enriched in chondrocyte 4 of NP-degenerative. (**B**) Violin plot showing GDF15, TRIB3 and TNFRSF10B are mainly highly enriched in chondrocyte 5 of NP-degenerative

**Fig. 7:**
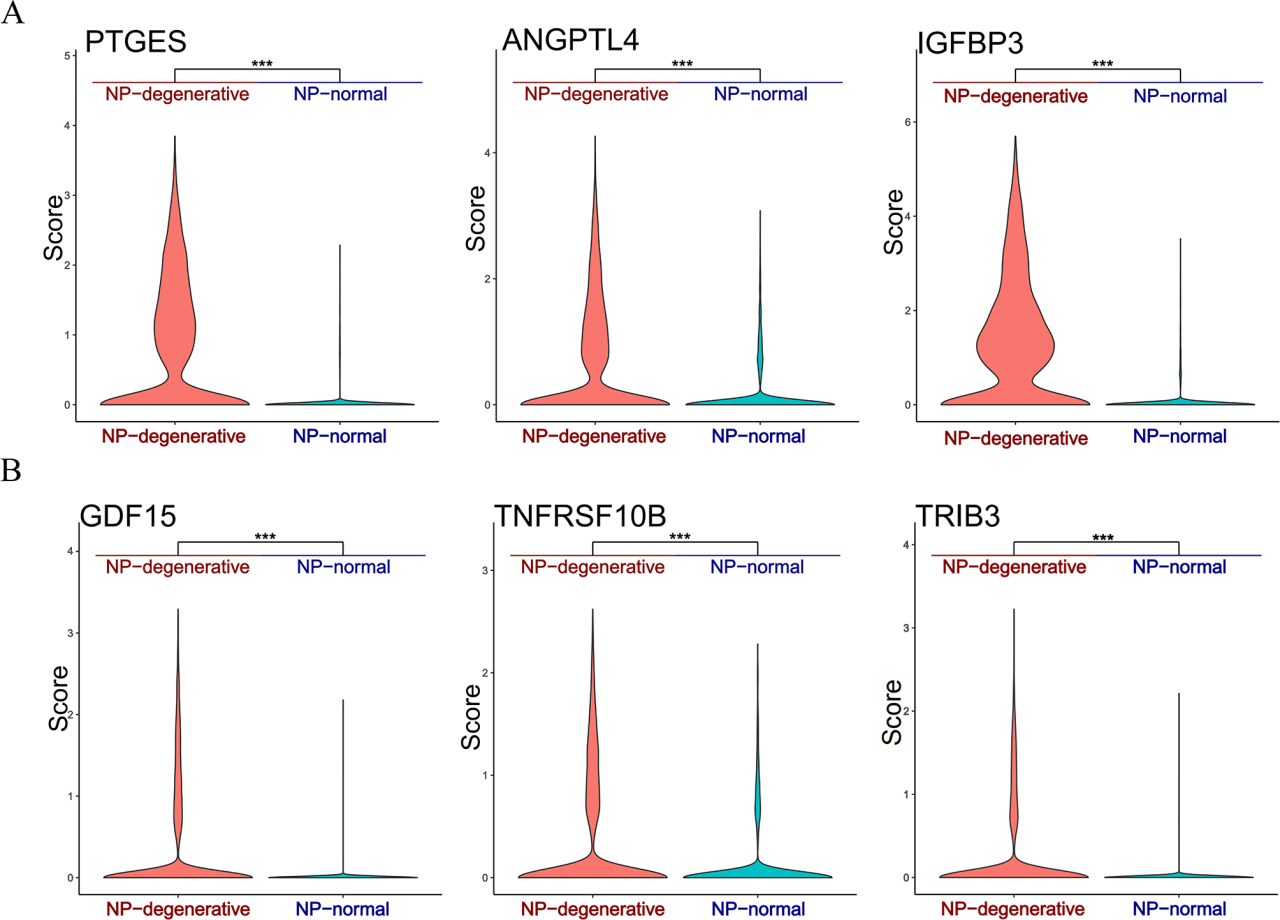
Expression of the marker genes. Differential expression analysis was performed comparing marker genes within NP-degenerative and NP-normal. Stars indicate significance level of gene expression difference between two samples by Wilcox test. *, *p* value < 0.05; **, *p* value < 0.01; ***, *p* value < 0.001

### scRNA-seq analysis reveals distinct marker genes in chondrocyte 4

Overall, chondrocyte 4 and chondrocyte 5 are higher proportions in NP-degenerative cells, but lesser proportions in NP-normal cells (Fig. 1C and D). In chondrocyte 4, some genes were identified that were predominantly expressed in NP degenerative cells, such as ANGPTL4, PTGES, and IGFBP3(Fig. 5A **and** Fig. 6A). And the expression is clearly different, Stars indicate significance level of gene expression difference between two samples by Wilcox test. ***, *P* value < 0.001(Fig. 7).

### scRNA-seq analysis reveals distinct marker genes in chondrocytes 5

Similarly, GDF15, TRIB3, and TNFRSF10B were found to be highly expressed in NP-degenerative cells following in-depth analysis of chondrocyte 5 (Fig. 5B **and** Fig. 6B). And the expression is clearly different, Stars indicate significance level of gene expression difference between two samples by Wilcox test. ***, *P* value < 0.001(Fig. 7).

### Expression of the marker genes within chondrocyte 4 and chondrocyte 5

**Fig. 8:**
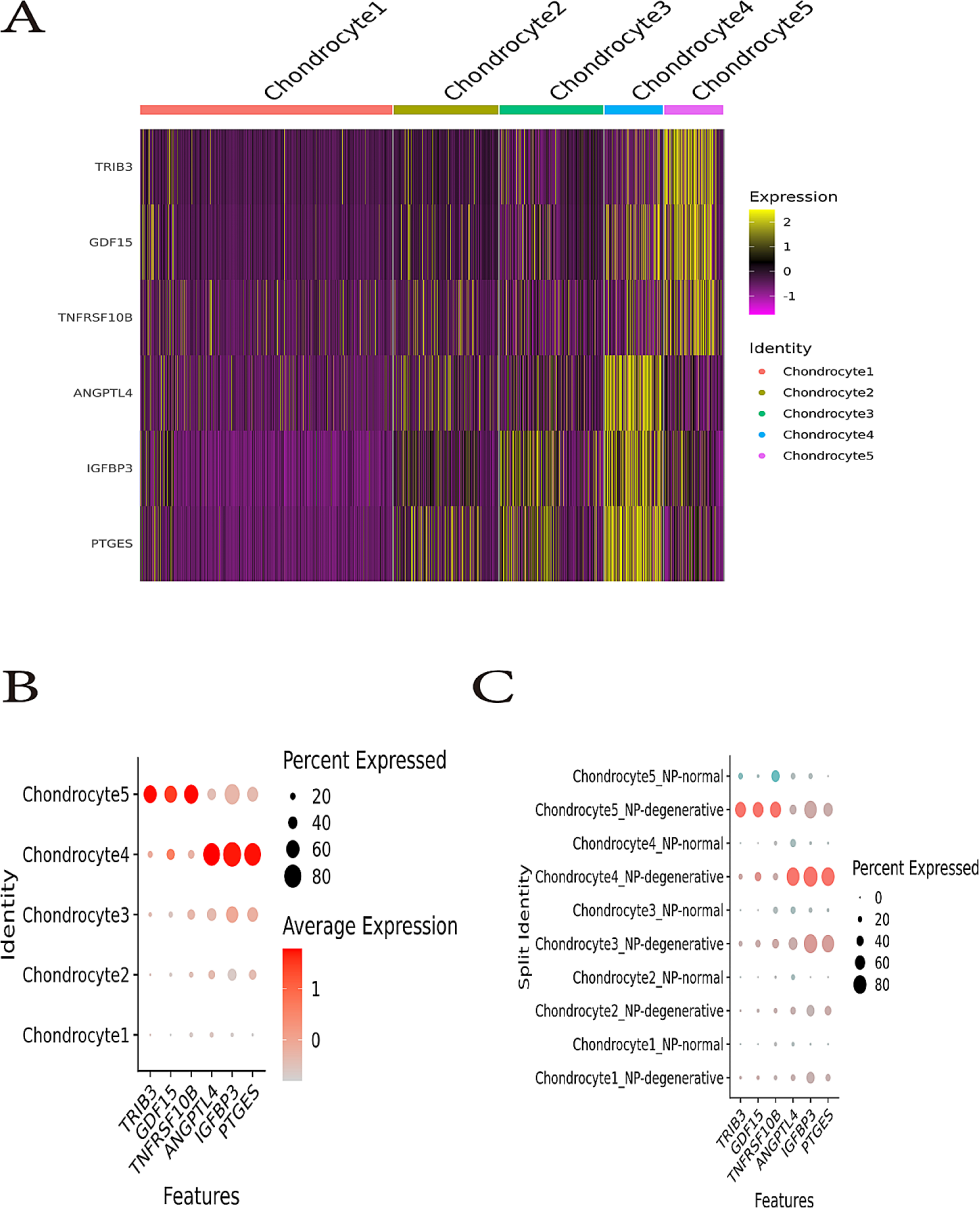
Expression of the marker genes. (**A**) Heatmap plot showing scaled expression of differentially expressed genes defining the five chondrocyte clusters. (**B**) GeneExp Bubble plot showing scaled expression of differentially expressed genes defining the five chondrocyte clusters. (**C**) Differential expression analysis was performed comparing marker genes within NP-degenerative and NP-normal

Heatmap plot showing scaled expression of differentially expressed genes defining the five chondrocyte clusters. Among these, ANGPTL4, PTGES, and IGFBP3 were mainly highly expressed in the chondrocytes 4 of NP-degenerative tissues. GDF15, TRIB3, and TNFRSF10B were mainly highly expressed in the chondrocytes 5 of the NP-degenerative tissues (Fig. 8A). Bubble plot also showing ANGPTL4, PTGES, and IGFBP3 are mainly highly expressed in the chondrocytes 4 of NP-degenerative tissues. GDF15, TRIB3, and TNFRSF10B were mainly highly expressed in the chondrocytes 5 of NP-degenerative tissues Fig. 8B **and** Fig. 8C).

### qRT-PCR

**Fig. 9:**
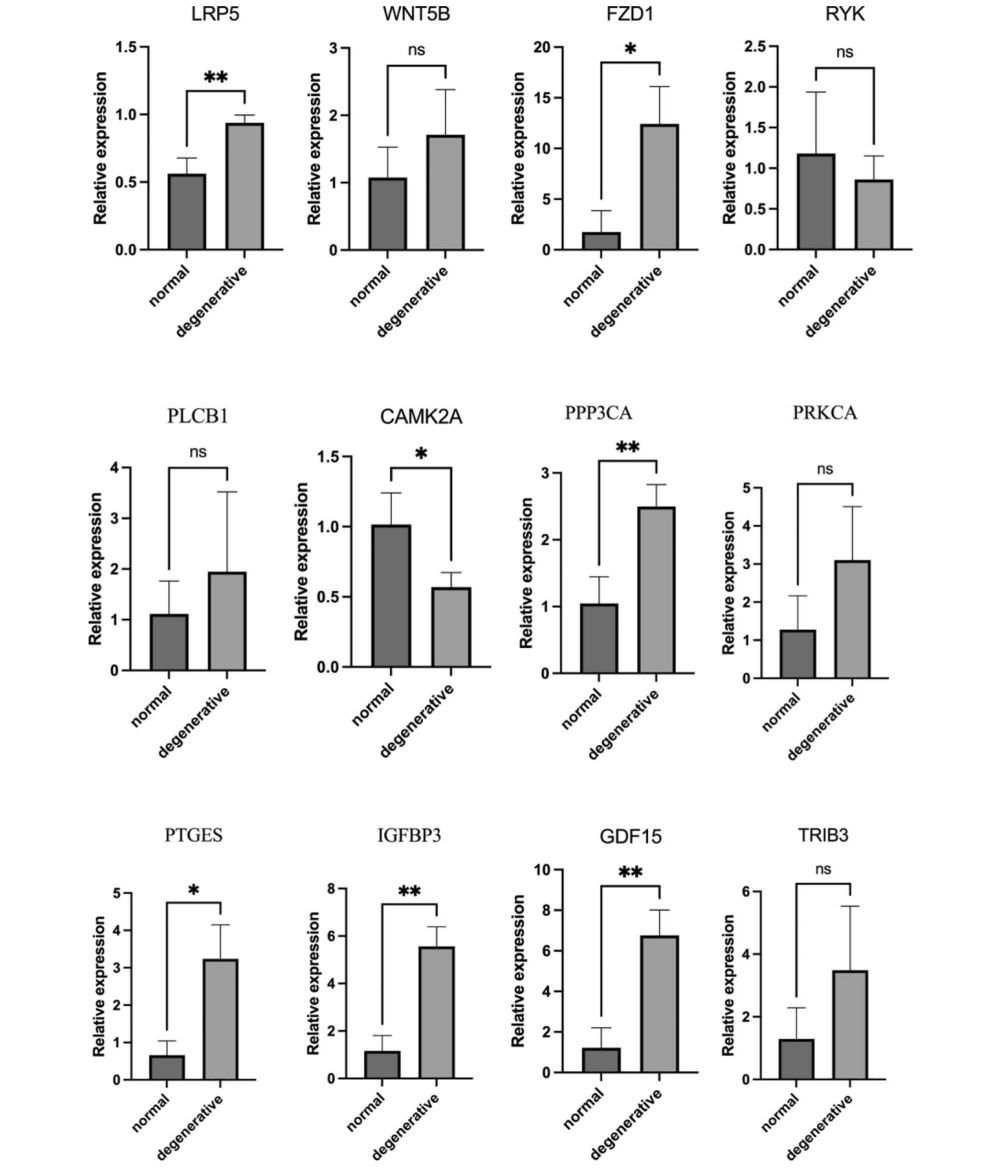
RT-qPCR was used to detect the mRNA expression of the genes expression in the Wnt/Ca^2+^ signaling pathway. *p < 0.05, *p < 0.01, *p < 0.001, *p < 0.0001

To confirm the genes expression in the Wnt/Ca^2+^ signaling pathway, we conducted in-vitro experiments. The results of qRT-PCR revealed that the expression of LRP5, FZD1, PPP3CA(CaN), PTGES, GDF15, IGFBP3 were significantly higher in degenerated nucleus pulposus tissues, and the difference was statistically significant (Fig. 9). However, probably due to changes in cell membrane receptors the expression levels of WNT5B and PLCB genes did not reach the expected results of the experiment (*p < 0.05,*p < 0.01,*p < 0.001,*p < 0.0001).

**Fig. 10:**
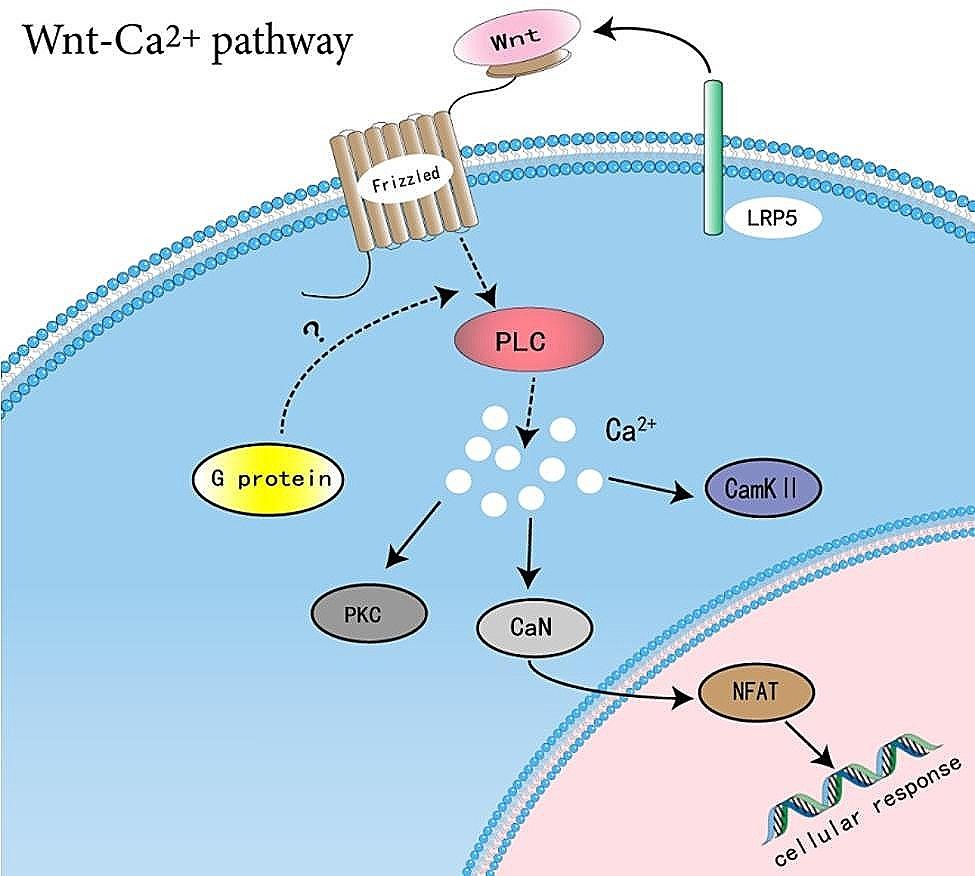
The schematic representation of Wnt/Ca^2+^ signaling pathway

## Discussion

The etiology of IVDD is complex, which pathogenesis remains incompletely understood. Aging, genetic factors and inflammation are all associated with the development and progression of IVDD [3]. Moreover, apoptosis, inflammation and degeneration of nucleus pulposus cells (NPC) are integral parts of IVDD [20]. The previous studies have demonstrated that various types of cells are involved in the process of IVDD, such as chondrocytes, endothelial cells, macrophages, neutrophils, and T cells, there are also close communication between them [16]. In recent years, genetically and regenerative stem cell-based therapies have intervened in the progress of IVDD. As the nucleus pulposus is the main tissue involved in the progression of IVDD, a better understanding of the transcriptional relationship of nucleus pulposus cells will improve the feasibility of clinical treatment [21]. Fernandes [22] recently reported the transcriptional landscape between NP and AF in healthy human discs by scRNA-seq. Unlike their strategy, in the present study, we focused more on the process of Wnt/Ca^2+^ signaling pathway in IVDD development and analyzed the changes among them through the transcriptome of scRNA-seq of NP cells. The Wnt/Ca^2+^ signaling pathway is an important branch of the Wnt signaling pathway, which signal transduction pathway is closely related to cellular responses caused by developmental, cancer and inflammatory stimuli [9]. Yang have shown that regulating related proteins in the Wnt/β-catenin signaling pathway can induce degeneration of nucleus pulposus cells and ultimately accelerate disc degeneration [10, 11]. The proposed vertebrate Wnt/Ca^2+^pathway. Activation of the Wnt/Ca^2+^ pathway results in intracellular Ca^2+^ release [23], and activation of the Ca^2+^-sensitive enzymes Ca^2+^-calmodulin-dependent protein kinase II (CamKII) and protein kinase C (PKC) in a b-catenin-independent manner [24, 25]. Although experimental data suggest a requirement for G proteins in this pathway, the question mark indicates that no direct interaction of G proteins with Frizzleds has been report [23–25]. Therefore, we performed scRNA-seq on more than 10,000 cells, then selected five chondrocyte populations within nucleus pulposus cells for analysis, and annotated the nature and frequency of NP cell clusters between groups. Our results provide the concept of human nucleus pulposus cell degeneration and Wnt/Ca^2+^ signaling pathway during IVDD development at the single-cell level, and provide several molecular proteins that may play an important role in the process of nucleus pulposus cell degeneration.

Firstly, we clustered the resulting cell data into five distinct clusters, chondrocyte 1, chondrocyte 2, chondrocyte 3, chondrocyte 4 and chondrocyte 5. As the main type of NP, chondrocytes play an important role in NP function. Therefore, we looked more closely at the differences between normal and degenerated nucleus pulposus cells in five chondrocyte ethnic groups. By comparison, we found that chondrocyte 1 had a higher percentage of NP-normal cells compared with NP-degenerative cells. In general, chondrocyte 4 and chondrocyte 5 accounted for a higher proportion in NP-degenerative, but a small proportion in NP-normal. Why are chondrocytes 4 and chondrocytes 5 highly represented in NP-degenerative and whether this is associated with the degeneration process of nucleus pulposus cells and intervertebral discs? This has aroused our great interest.

Subsequently, in a visual analysis of the five different chondrocyte classes described above, we found that proteins associated with the Wnt/Ca^2+^ pathway, such as wnt5B, FZD1, PLC (PLCB1), CaN (PPP3CA), NAFATC1, all of which accounted for macroscopically different ratios in the five distinct clusters in NP-degenerative cells and NP-normal cells. Precisely, although Wnt5B is present in NP-degenerative and NP-normal cells, Wnt5B is mainly present in chondrocyte 3, chondrocyte 4 and chondrocyte 5 in NP-degenerative cells compared with NP-normal cells, and less in the other chondrocytes. At the same time, we have identified LRP5, low-density lipoprotein receptor-related protein 5 (LRP5), a receptor that has been demonstrated to be involved in activating Wnt signaling [26, 27], and is involved in monocyte differentiation to macrophages and apoptosis [28]. In addition, it has been shown that by activating the canonical Wnt/β-catenin signaling pathway, can induce the synthesis of various proteins such as Rac1 [10], Periostin [11], TGF-β, and insulin growth factor [12] in disc cells, thereby accelerating disc degeneration. However, signaling pathways and their key proteins in degenerative NP cells have not been fully elucidated, and little or no Wnt/Ca^2+^ signaling pathway has been investigated in human degenerative NP cells. The Wnt/Ca^2+^ signaling pathway is an important branch of the Wnt signaling pathway, and its signal transduction pathway is closely related to cellular responses caused by developmental, cancer, and inflammatory stimuli [9]. Noncanonical Wnt/Ca^2+^ signaling leads to transient increases in cytosolic free calcium through G proteins and phospholipases, reactivates the phosphatase calcineurin, induces cytosolic NFAT phosphorylation to bind AP1, and finally initiates transcription of target genes [28]. Therefore, we came to the following conclusions: as a co-receptor of members of the seven-transmembrane frizzled family, WNT5B activated by LRP5, which promotes apoptosis and inflammatory responses in nucleus pulposus cells by regulating the Wnt/Ca^2+^signaling pathway, thereby promoting disc degeneration(Fig. 10). Moreover, this process is closely related to the abundance of chondrocyte 4 and chondrocyte 5 in NP-degenerative cells. Furthermore, LRP5 is also highly expressed in chondrocyte 4 and chondrocyte 5 of NP-degenerative cells and especially in chondrocytes 5. This fits well with our conjecture because related proteins in the Wnt/Ca^2+^ pathway, such as wnt5B, FZD1, PLC (PI-PLB1), CaN (PPP3CA) and NAFATC1 are similarly expressed.

To investigate the relationship between Wnt/Ca^2+^ signaling pathway and apoptosis, inflammation in nucleus pulposus chondrocytes, we extracted their representative genetic markers from the raw data for further analysis. In chondrocyte 4, we found a number of related genes mainly highly expressed in NP-degenerative cells, which were ANGPTL4, PTGES and IGFBP3. ANGPTL4 acts as a serum hormone to regulate insulin sensitivity [29], and this protein can be an apoptotic survival factor in vascular endothelial cells [30]. Prostaglandin E synthase (PTGES) plays a critical role (by similarity) in inflammation, fever and pain [31]. The gene expression of insulin-like growth factor binding protein 3 (IGFBP3) is closely related to apoptosis, autophagy and cellular senescence in nucleus pulposus cells [32]. Similarly, in chondrocytes 5, we also found a number of related genes that were mainly highly expressed in NP-degenerative cells, which were GDF15, TRIB3 and TNFRSF10B. Among them, growth and differentiation factor 15 (GDF15) is an inflammation-related hormone [33], and GDF15 is also closely related to apoptosis [34]. Trilobar pseudokinase 3 (TRIB3) is a critical determinant of many cellular processes, including apoptosis [35]. TNF receptor superfamily member 10b (TNFRSF10B) encodes a protein that is a member of the TNF receptor superfamily, and TNFRSF10B expression is closely related to mechanisms associated with apoptosis [36]. Therefore, we came to the conclusions: as a co-receptor of members of the seven-transmembrane frizzled family, WNT5B activated by LRP5 [37], which promotes apoptosis and inflammatory responses in nucleus pulposus cells by regulating the Wnt/Ca^2+^signaling pathway, thereby promoting disc degeneration. Some genes are widespread in chondrocyte 4, and chondrocyte 5 may play an important role in the process of lumbar disc degeneration together with the Wnt/Ca^2+^signaling pathway, such as ANGPTL4, PTGES, IGFBP3, GDF15, TRIB3, and TNFRSF10B.

## Conclusions

This study systematically dissected normal and degenerative nucleus pulposus tissues in human at the single-cell level. Single-cell RNA sequencing of nucleus pulposus cells revealed differential expression of the Wnt/Ca^2+^ signaling pathway in normal and degenerative nucleus pulposus cells, and this differential expression may be closely related to the enrichment of chondrocyte 4 and chondrocyte 5 in degenerative nucleus pulposus cells. In degenerative nucleus pulposus cells, LRP5 activates Wnt5B, which promotes apoptosis and inflammatory responses in nucleus pulposus cells by regulating the Wnt/Ca^2+^ signaling pathway, thereby promoting disc degeneration. Six proteins, including ANGPTL4, PTGES, IGFBP3, GDF15, TRIB3, and TNFRSF10B, may be closely associated with nucleus pulposus cell apoptosis and inflammatory responses. ANGPTL4, IGFBP3, PTGES in chondrocytes 4 and TRIB3, GDF15, TNFRSF10B in chondrocytes 5 may play an important role in the lumbar disc degeneration process.

Further confirmation of the relationship between Wnt/Ca^2+^ signaling pathway and related proteins and degeneration of nucleus pulposus cells is still needed in the future, but our current work remains of some value for understanding the progression of IVDD. Both transcriptome analysis of NP at single cell resolution and identification of novel chondrocyte clusters have facilitated the understanding of IVDD. In particular, the differential analysis of the Wnt/Ca^2+^ signaling pathway in normal and degenerative nucleus pulposus cells, as well as the six related proteins found, in-depth study of this information may open a new window for future therapeutic intervention in IVDD.

## Data Availability

Supplemental material for this article can be found at https://www.ncbi.nlm.nih.gov/geo/query/acc.cgi?acc=GSE205535.

## References

[1] Zheng H, Wang T, Li X, He W, Gong Z, Lou Z (2020). LncRNA MALAT1 exhibits positive effects on nucleus pulposus cell biology in vivo and in vitro by sponging miR-503. BMC Mol Cell Biol.

[2] Vergroesen PPA, Kingma I, Emanuel KS, Hoogendoorn RJW, Welting TJ, van Royen BJ (2015). Mechanics and biology in intervertebral disc degeneration: a vicious circle. Osteoarthr Cartil.

[3] Feng Y, Egan B, Wang J (2016). Genetic factors in intervertebral disc degeneration. Genes Dis.

[4] Hoy D, Brooks P, Blyth F, Buchbinder R (2010). The epidemiology of low back pain. Best Pract Res Clin Rheumatol.

[5] Tao XZ, Jing L, Li JH (2018). Therapeutic effect of transforaminal endoscopic spine system in the treatment of prolapse of lumbar intervertebral disc. Eur Rev Med Pharmacol Sci.

[6] Loreto C, Musumeci G, Castorina A, Loreto C, Martinez G (2011). Degenerative disc disease of herniated intervertebral discs is associated with extracellular matrix remodeling, vimentin-positive cells and cell death. Ann Anat.

[7] Li Z, Yu X, Shen J, Chan MTV, Wu WKK (2015). MicroRNA in intervertebral disc degeneration. Cell Prolif.

[8] Jiang L, Zhang X, Zheng X, Ru A, Ni X, Wu Y (2013). Apoptosis, senescence, and autophagy in rat nucleus pulposus cells: implications for diabetic intervertebral disc degeneration. J Orthop Res.

[9] De A (2011). Wnt/Ca^2+^ signaling pathway: a brief overview. Acta Biochim Biophys Sin.

[10] Yang X, Sun Y, Li X, Zhang W (2022). Rac1 regulates nucleus pulposus cell degeneration by activating the Wnt/β-catenin signaling pathway and promotes the progression of intervertebral disc degeneration. Am J Physiol Cell Physiol.

[11] Zhu D, Wang Z, Zhang G, Ma C, Qiu X, Wang Y (2022). Periostin promotes nucleus pulposus cells apoptosis by activating the Wnt/β-catenin signaling pathway. FASEB J.

[12] Chen J, Jia YS, Liu GZ, Sun Q, Zhang F, Ma S (2017). Role of LncRNA *TUG1* in intervertebral disc degeneration and nucleus pulposus cells via regulating Wnt/β-catenin signaling pathway. Biochem Biophys Res Commun.

[13] Zhao X, Cui DJ, Yuan WQ, Chen C, Liu Q (2022). Berberine represses Wnt/β-catenin pathway activation via modulating the microRNA-103a-3p/Bromodomain-containing protein 4 axis, thereby refraining pyroptosis and reducing the intestinal mucosal barrier defect induced via colitis. Bioengineered.

[14] Potter SS (2018). Single-cell RNA sequencing for the study of development, physiology and disease. Nat Rev Nephrol.

[15] Schelker M, Feau S, Du J, Ranu N, Klipp E, MacBeath G (2017). Estimation of immune cell content in tumour tissue using single-cell RNA-seq data. Nat Commun.

[16] Li Z, Ye D, Dai L, Xu Y, Wu H, Luo W (2022). Single-cell RNA sequencing reveals the difference in human normal and degenerative nucleus pulposus tissue profiles and cellular interactions. Front Cell Dev Biol.

[17] Chen S, Zhou Y, Chen Y, Gu J (2018). Fastp: an ultra-fast all-in-one FASTQ preprocessor. Bioinformatics.

[18] Smith T, Heger A, Sudbery I (2017). UMI-tools: modeling sequencing errors in Unique Molecular identifiers to improve quantification accuracy. Genome Res.

[19] Shaath H, Vishnubalaji R, Elkord E, Alajez NM (2020). Single-cell transcriptome analysis highlights a role for neutrophils and inflammatory macrophages in the pathogenesis of severe COVID-19. Cells.

[20] Sun K, Zhu J, Yan C, Li F, Kong F, Sun J (2021). CGRP regulates nucleus pulposus cell apoptosis and inflammation via the MAPK/NF-κB signaling pathways during intervertebral disc degeneration. Oxid Med Cell Longev.

[21] Zhang Y, Han S, Kong M, Tu Q, Zhang L, Ma X (2021). Single-cell RNA-seq analysis identifies unique chondrocyte subsets and reveals involvement of ferroptosis in human intervertebral disc degeneration. Osteoarthr Cartil.

[22] Fernandes LM, Khan NM, Trochez CM, Duan M, Diaz-Hernandez ME, Presciutti SM (2020). Single-cell RNA-seq identifies unique transcriptional landscapes of human nucleus pulposus and annulus fibrosus cells. Sci Rep.

[23] Slusarski DC, Corces VG, Moon RT (1997). Interaction of wnt and a frizzled homologue triggers G-protein-linked phosphatidylinositol signalling. Nature.

[24] Kühl M, Sheldahl LC, Malbon CC, Moon RT (2000). Ca^2+^/calmodulin-dependent protein kinase II is stimulated by wnt and frizzled homologs and promotes ventral cell fates in *Xenopus*. J Biol Chem.

[25] Sheldahl LC, Park M, Malbon CC, Moon RT (1999). Protein kinase C is differentially stimulated by wnt and frizzled homologs in a G-protein-dependent manner. Curr Biol.

[26] Hay E, Faucheu C, Suc-Royer I, Touitou R, Stiot V, Vayssière B (2005). Interaction between LRP5 and Frat1 mediates the activation of the wnt canonical pathway. J Biol Chem.

[27] Borrell-Pages M, Vilahur G, Romero JC, Casaní L, Bejar MT, Badimon L (2016). LRP5/canonical wnt signalling and healing of ischemic myocardium. Basic Res Cardiol.

[28] Scholz B, Korn C, Wojtarowicz J, Mogler C, Augustin I, Boutros M (2016). Endothelial RSPO3 controls vascular stability and pruning through non-canonical WNT/Ca^2+^/NFAT signaling. Dev Cell.

[29] Xu A, Lam MC, Chan KW, Wang Y, Zhang J, Hoo RLC (2005). Angiopoietin-like protein 4 decreases blood glucose and improves glucose tolerance but induces hyperlipidemia and hepatic steatosis in mice. Proc Natl Acad Sci U S A.

[30] Shibata K, Nakayama T, Hirakawa H, Hidaka S, Nagayasu T (2010). Clinicopathological significance of angiopoietin-like protein 4 expression in oesophageal squamous cell carcinoma. J Clin Pathol.

[31] Murakami M, Naraba H, Tanioka T, Semmyo N, Nakatani Y, Kojima F (2000). Regulation of prostaglandin E_2_ biosynthesis by inducible membrane-associated prostaglandin E_2_ synthase that acts in concert with cyclooxygenase-2. J Biol Chem.

[32] Chen G, Zhou X, Xu Z (2019). Retracted: effects of IGFBP3 gene silencing mediated inhibition of ERK/MAPK signaling pathway on proliferation, apoptosis, autophagy, and cell senescence in rats nucleus pulposus cells. J Cell Physiol.

[33] Luan HH, Wang A, Hilliard BK, Carvalho F, Rosen CE, Ahasic AM (2019). GDF15 is an inflammation-induced central mediator of tissue tolerance. Cell.

[34] Xu G, Chen J, Jo S, Grayson TB, Ramanadham S, Koizumi A (2022). Deletion of GDF15 reduces ER stress-induced beta-cell apoptosis and diabetes. Endocrinology.

[35] Lin RJ, Wu I, Hong JY, Liu BH, Liang RY, Yuan TM (2018). Capsaicin-induced TRIB3 upregulation promotes apoptosis in cancer cells. Cancer Manag Res.

[36] Li T, Su L, Lei Y, Liu X, Zhang Y, Liu X (2015). DDIT3 and KAT2A proteins regulate TNFRSF10A and TNFRSF10B expression in endoplasmic reticulum stress-mediated apoptosis in human lung cancer cells. J Biol Chem.

[37] Borrell-Pagès M, Romero JC, Badimon L (2014). LRP5 negatively regulates differentiation of monocytes through abrogation of wnt signalling. J Cell Mol Med.

